# Metal oxide nanostructures by a simple hot water treatment

**DOI:** 10.1038/s41598-017-07783-8

**Published:** 2017-08-02

**Authors:** Nawzat S. Saadi, Laylan B. Hassan, Tansel Karabacak

**Affiliations:** Department of Physics and Astronomy, University of Arkansas at Little Rock 2801 South University Avenue, Little Rock, AR 72204 USA

## Abstract

Surfaces with metal oxide nanostructures have gained considerable interest in applications such as sensors, detectors, energy harvesting cells, and batteries. However, conventional fabrication techniques suffer from challenges that hinder wide and effective applications of such surfaces. Most of the metal oxide nanostructure synthesis methods are costly, complicated, non-scalable, environmentally hazardous, or applicable to only certain few materials. Therefore, it is crucial to develop a simple metal oxide nanostructure fabrication method that can overcome all these limitations and pave the way to the industrial application of such surfaces. Here, we demonstrate that a wide variety of metals can form metal oxide nanostructures on their surfaces after simply interacting with hot water. This method, what we call hot water treatment, offers the ability to grow metal oxide nanostructures on most of the metals in the periodic table, their compounds, or alloys by a one-step, scalable, low-cost, and eco-friendly process. In addition, our findings reveal that a “plugging” mechanism along with surface diffusion is critical in the formation of such nanostructures. This work is believed to be of importance especially for researchers working on the growth of metal oxide nanostructures and their application in functional devices.

## Introduction

As nanomaterials find new applications in various fields, metal oxide nanostructures (MONSTRs) are not an exemption due to their exceptional physical and chemical properties. Recently, MONSTRs have been used in proof-of-concept electronic^1–5^, optical^6–8^, and sensing^9–14^ devices; yet, their synthesis techniques endure several hurdles that limit their large-scale production. So far, various approaches have been used to synthesize metal oxide nanostructures, including chemical vapor deposition^15–17^, thermal evaporation^18–20^, hydrothermal^21–23^, and laser ablation^24, 25^. Each of these methods incorporates challenging aspects such as being limited to only a few number of materials, costly processing or demanding high temperatures, which have limited their commercial applications. Therefore, fabricating MONSTRs using a new alternative method with practical features including applicability to wide range of materials, high-throughput fabrication, catalyst-free growth, low-synthesis temperature, low-cost equipment, and being environment-friendly is highly desirable in order to overcome the limitations of conventional techniques. Recently, a simple hot water treatment (HWT) process has been demonstrated to produce MONSTRs of aluminum oxide (Al_2_O_3_)^26^, zinc oxide (ZnO)^27, 28^, and copper oxide (CuO)^29, 30^. There also has been recent reports on using water vapor for low temperature oxidation^31^ and crystallization^32^ of nanostructured metals and oxides, respectively. In the 1970s, the HWT technique had been used to produce metal oxide thin films mainly with the aim of adhesion improvement between metal joints^33^; yet there has been no report on MONSTR formation until recently. HWT overall involves immersing a metal substrate into a hot deionized (DI) water at temperatures typically ≥75 °C. On the other hand, this simple technique has been overlooked for its potential capability to form MONSTRs in a scalable, low-cost, and eco-friendly manner. In this study, we show that HWT can form MONSTRs on most of the metals in the period table as well as some of their alloys and compounds. In addition, growth mechanisms of MONSTRs formation have been investigated in detail that revealed a new “plugging” process that involves migration of metal oxide molecules through water and deposition to farther locations on the surface. The results of this work are believed to pave way to numerous applications of metal oxide nanostructures produced by a facile HWT method and fundamental understanding of the growth process involved.

When an oxide-free surface of a metal is immersed in water, metal atoms at the solid/liquid interface tend to form cations, which can be easily oxidized to a higher oxidation state^34, 35^. For most metals except noble ones, the oxide formed is thermodynamically more stable than the elemental metal in water. Therefore, the metal surface will oxidize and will be covered by metal oxide when immersed in water^36, 37^. The formed metal oxide typically creates a compact (non-porous) continuous oxide layer, known as a thin film metal oxide, on the surface of a metal and the formation of such an oxide film has been widely reported as well as the growth mechanism involved^35, 37^. However, most of the previous studies focused on metal oxide formation in water at room temperature conditions. Therefore, we investigated the possibility of MONSTR formation at elevated temperatures using HWT.

## Results and Discussion

**Figure 1:**
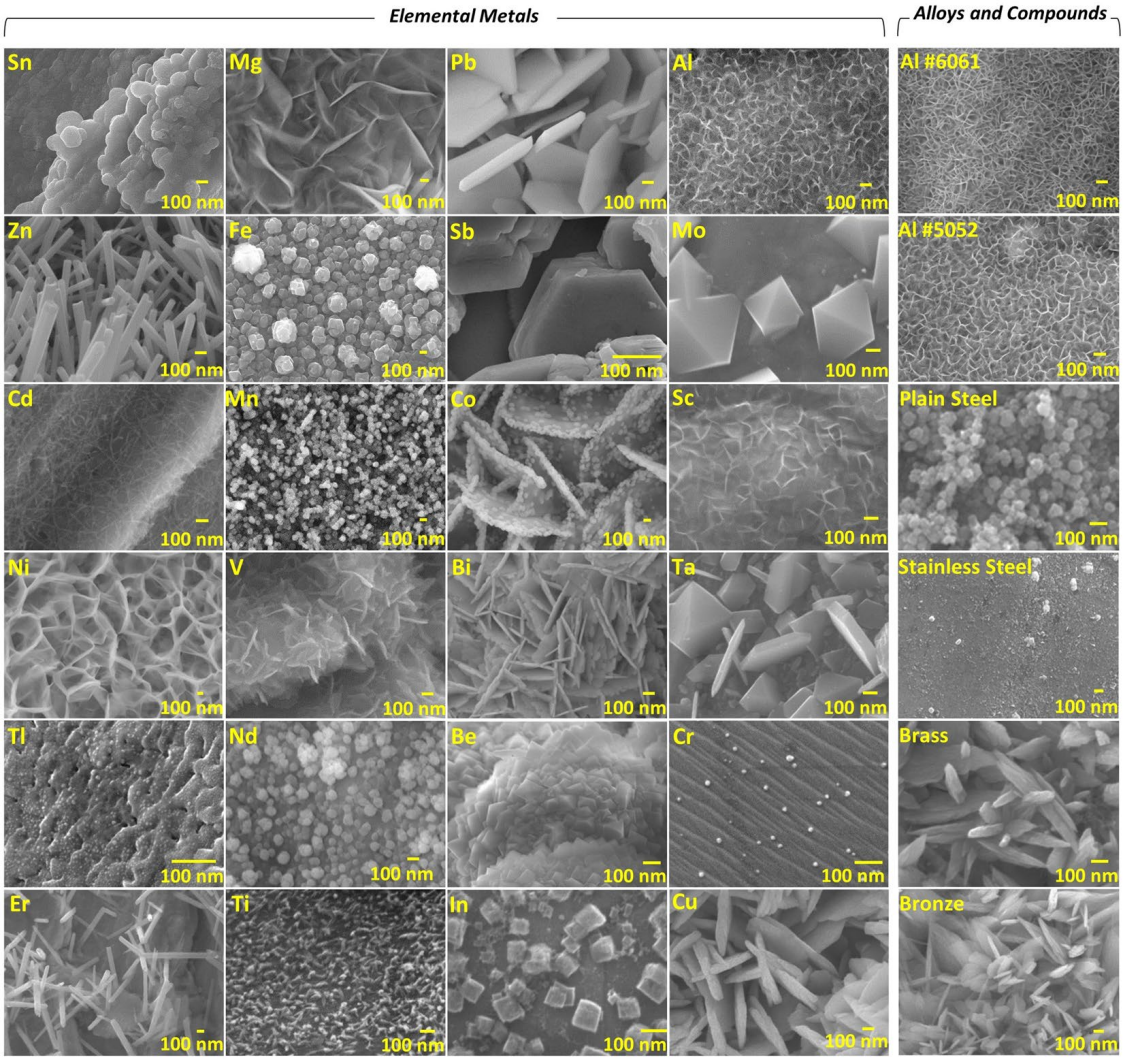
Top-view SEM images of various elemental metals and some alloys and metallic compounds after hot water treatment (HWT) are shown. Sequence of the images are arranged based on the treatment time required in order to form well-defined metal oxide nanostructures (MONSTRs): from shortest to the longest treatment time in the order of (**a**) from left to the right and top to the bottom (**b**) from top to the bottom.

**Figure 2:**
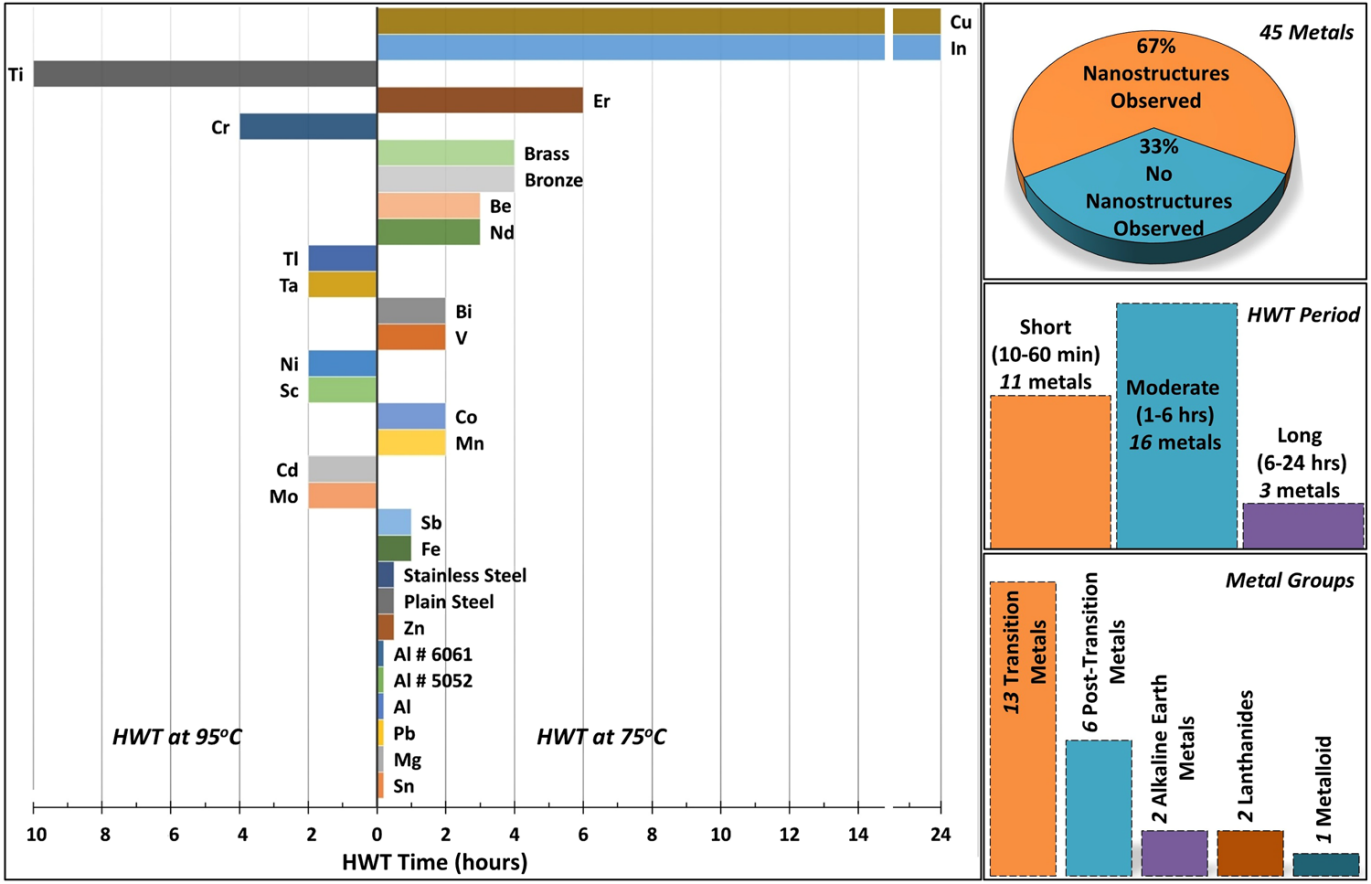
Bar diagram illustrates approximate minimum HWT times required before distinct MONSTRs can be observed on the metals of this study. Most of the metals form nanostructures at 75 °C (pointing to the right in bar diagram) while few others require a higher temperature of 95 °C (left). On the right column, some statistical data about the response of metals to HWT with respect to metal group (top histogram graph), required treatment periods for nanostructure formation (middle), and percentage of metals that did or did not respond to HWT (bottom).

In this study, 45 different types of metals that involve several pure elements, alloys, and compounds were treated by hot DI water in a search for which metals respond to HWT and form MONSTRs. We first investigated the change in morphology of metal surfaces after HWT was conducted at a water temperature of 75 °C. Those metals that did not form MONSTRs were treated at longer times, and some at 95 °C. Figure 1 shows the scanning electron microscopy (SEM) images of those metals that formed MONSTRs on the surface, which corresponds to 30 out of 45 metals studied. In other words, MONSTRs were able to grow on the surface of about 67% of all metals of this work, which is illustrated in the pie chart of Fig. 2. Most of the nanostructures observed in Fig. 1 were confirmed to be metal oxides of thermodynamically stable compositions as evidenced by energy dispersive X–ray spectroscopy (EDS, Table S1) and X-ray diffraction (XRD) analysis (Fig. S1). It is believed that metal hydroxide phase that could form through hydrolysis, which is typically amorphous^38^, is insignificant during the growth of HWT MONSTRs mainly due to the notable hydrogen bubble formation observed for most of the metals studied (e.g. supplementary video 1 for Al) and well defined textured shapes of the MONSTR crystals (Fig. 1). Images in Fig. 1 were sequenced in the order based on the treatment times required to form well-defined metal oxide nanostructures. For example, tin (Sn) forms MONSTRs after 10 min at 75 °C (Fig. 1, elements group, upper left), while it took 24 hrs for copper (Cu; Fig. 1, elements group, bottom right). Among the alloys and compounds investigated, aluminum alloys produced MONSTRs at 10 mins and bronze formed nanostructures only after 7 hrs at 75 °C. No MONSTRs were observed at 75 °C for few of the metals even after 24 hrs. These samples were then treated at 95 °C which resulted in MONSTR formation. For instance, nanostructures were observed on indium (In) at 95 °C after about 10 hrs. Depending on the metal used, several MONSTR shapes can be observed in Fig. 1 including cubes, pyramids, plates, wires, spheres, and leaf-like nanostructures. As will be discussed below, such different geometries are believed to originate from different crystal structures, surface diffusion, and surface energy minimization through the formation of certain crystal facets^39^. In addition, some metals (e.g. Al, Mg, Ni) formed interconnected MONSTRs, while for others they were well-isolated (e.g. Zn, Mo, In, Cu). The MONSTRs in all of the metals studied uniformly coated substrates (Fig. S2) that indicate the scalability of HWT technique.

Although the formation of metal oxide thin film at the metal/water interface during HWT has been widely reported^33, 40–44^, for the first time, our results reveal that a wide range of metals can also form MONSTRs instead of thin films by HWT. One possible reason why such a simple process of nanostructure formation has been overlooked might be due to the focus of earlier studies on metal oxide composition more than the change in morphology. Another reason might be due to the sample preparation process in those studies, which might not have removed the native oxide on the surface before HWT that can act as a self-diffusion barrier^45^ and hinder the MONSTR formation. As we will discuss below, growth mechanisms of MONSTR formation during HWT can strongly depend on the existence of non-oxidized metal atoms at the water/metal interface. We also note that remaining 15 metals out of the 45 that did not respond to HWT at the conditions used in our experiments might still form MONSTRs at longer treatment times or higher temperatures, which needs further investigation.

The bar diagram in Fig. 2 (left) summarizes the temperature values and critical treatment times used in order to form MONSTRs on the surface of those 30 metals that responded to HWT. As can be seen, 22 of the 30 metals were able to form MONSTRs at the lower temperature of 75 °C, while the remaining 8 required 95 °C. This shows the potential of HWT process as a low temperature method to synthesize metal oxide nanostructures. Also, as illustrated in the histogram of Fig. 2 (center right), most of the metals studied produced MONSTRs within only a few hours of the treatment time, which makes HWT as a good candidate for the high throughput fabrication method. Especially, the metals that are important for industrial applications such as Al, Zn, Mg, Pb, Al alloys, and steels, formed MONSTRs even within tens of minutes.

**Figure 3:**
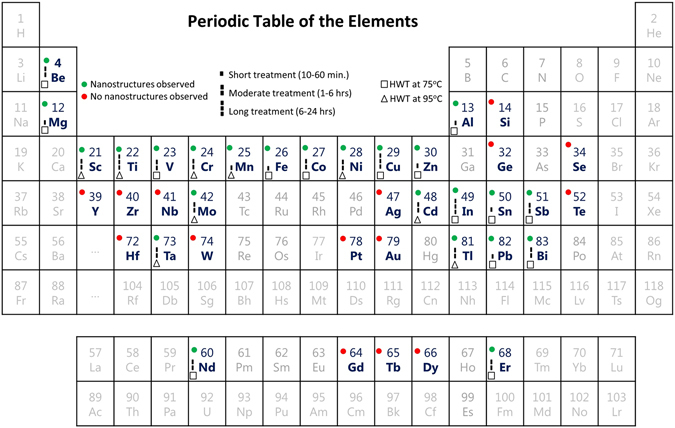
Periodic table of elements showing the elemental metals included in this study (bold blue font) and their response to HWT time and temperature. Green solid circles refer to the metals which responded to the HWT process and MONSTRs were observed; while red solid circles correspond to the metals that did not shows any sign of MONSTR formation. Treatment temperatures are represented by square shapes for HWT treatment at 75 °C and triangles shape for 95 °C. Treatment time is denoted by solid bars.

In addition, as can be observed in Fig. 3 and metal-groups histogram in Fig. 2, transition, post-transition and alkaline metals seem to be more reactive to HWT compared to the other groups as expected from the general behavior of metal-water reaction^46^. Among transition metals covered by this study (21 metals), 13 of them (Sc, Ti, V, Mn, Cr, Fe, Co, Ni, Cu, Zn, Mo, Cd, and Ta) responded to HWT and MONSTRs were observed, while the others (Zr, Y, Nb, Ag, Hf, W, Pt, and Au) did not show any notable response. For example, HWT at 95 °C for 36 hours was performed on Pt and W, yet no morphological or compositional changes were detected when characterized by SEM, XRD, and EDS. Except for Ga, which melts at room temperature and was excluded from this study, the surface of all post-transition metals (Al, In, Tl, Sn, Pb and Bi) showed the formation of nanoscale features after HWT. Also, Mg and Be from the alkaline earth metal group were included in this study and MONSTRs were observed on their surfaces after HWT. Amid the lanthanides investigated (Nd, Gd, Tb, Dy, and Er), only Nd and Er responded to the HWT process. Finally, only Sb from the metalloids studied (B, Si, Ge, As, Sb, and Te) showed the formation of MONSTRs after HWT.

**Figure 4:**
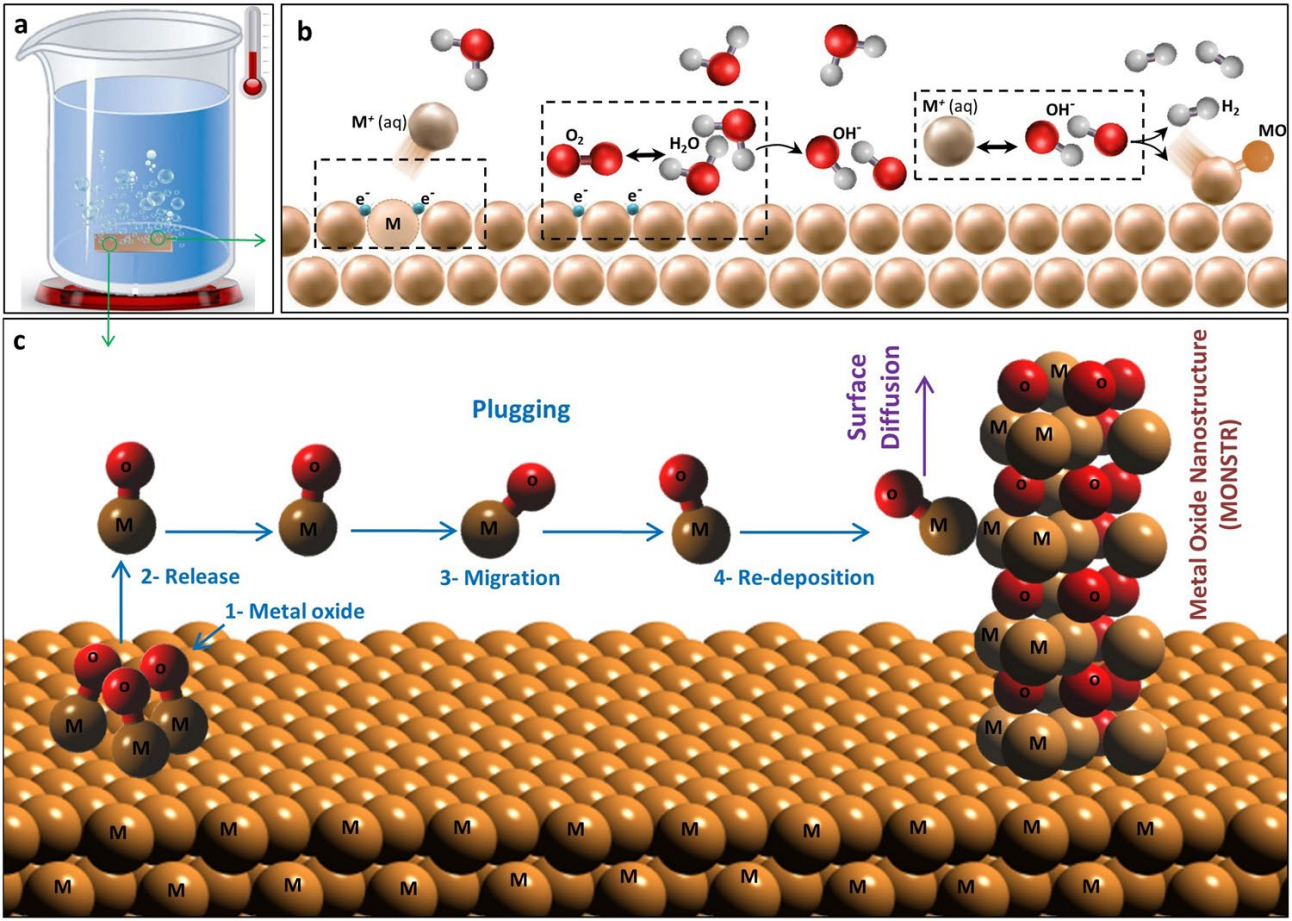
HWT process and steps involved in the formation of MONSTRs. (**a**) HWT process, (**b**) metal oxide formation during HWT at metal/water interface, and (**c**) steps involved in the formation of MONSTR during HWT are illustrated. Main nanostructure formation mechanism includes “plugging” and surface diffusion.

Furthermore, we investigated the potential growth mechanisms involved in the formation of MONSTRs. Formation of planar metal oxide films in water is widely reported in the literature^47–51^ (Note: As discussed above, metal hydroxide formation is believed to be insignificant in our work). As shown in Fig. 4b, the process starts with the formation of a positively charged metal ion that gets released into the water, leaving electrons behind on the surface. This metal cation still stays close to the water/metal interface due to the negative potential of the solid surface. Then, electrons on the surface can react with adsorbed oxygen and water molecules to produce hydroxyl ions. Finally, metal ions in water can react with hydroxyl ions on the surface to form a metal oxide molecule along with hydrogen^48^. Hydrogen is released and oxide molecules can form a flat metal oxide film, but this mechanism alone cannot explain the metal oxide nanostructures observed in HWT. Here, we propose a new growth mechanism that can lead to the formation of MONSTRs by HWT.

Figure 4c illustrates the important steps of MONSTR formation during HWT. The first step involves the formation of metal oxide molecules on the surface of a metallic substrate (“#1 Metal Oxide” in Fig. 4), which follows a reaction similar to the steps of metal oxide film formation described above (Fig. 4b). In our HWT experiments, we observed hydrogen gas formation that further supports this first step (supplementary video 1). As a potential next step during MONSTR growth, metal oxide molecules can diffuse on the surface. However, surface diffusion is not believed to be the dominant mechanism that explains the nanostructure formation at the relatively low temperature conditions of HWT (<100 °C). A more likely next step is a dissolution-precipitation process called “plugging”^52^ that has been used to describe the corrosion of metals. It involves the release of the metal oxide molecule (“#2 Release”) from the surface into the liquid followed by transportation through water (“#3 Migration”) and precipitation (“#4 Re-Deposition”) onto another surface position. Re-deposited molecules can initiate the formation of isolated nanostructures. However, the random nature of plugging might not be sufficient to explain smooth crystalline surfaces observed in HWT nanostructures. The re-deposited metal oxide molecules can diffuse on the surface, which may help in forming smooth individual nanostructures observed in the SEM images of Fig. 1.

These individual steps may be dependent on several important factors. For example, in the metal oxidation stage, oxidation rate can depend on the detailed water chemistry such as pH level, conductivity, and dissolved oxygen^53^. It can also depend metal-specific properties including ionization energy, electronegativity, charge/size ratio, and energy of oxide formation, which all can play critical roles in the responsivity of a given metal in HWT in forming MONSTRs. However, we could not observe a clear correlation between any of these individual parameters with the critical reaction times observed in the bar diagram of Fig. 2. This indicates that the limiting intermediate reaction during metal oxide formation varies from material to material. After the oxidation step, when the adhesion forces between a metal oxide molecule and metal substrate are weak or there is a liquid movement, the release step can become easier and make the plugging process more dominant. Especially in the case of low surface diffusion rates, such plugging process can lead to the formation of fractal-like rough nanostructures. Also, initial substrate surface chemistry can play a critical role in the nucleation of metal oxide nanostructures. It is believed that metal oxide molecules may preferentially stick to defect sites (e.g., voids or grain boundaries with dangling bonds) that can act as nucleation regions. Migration and re-deposition may also depend on external factors such as liquid flow patterns, substrate morphology, mechanical vibrations, or even external magnetic and electric fields.

Extra evidence of the migration and re-deposition steps taking place in HWT is shown by simultaneously placing Zn and Cu plates separated from each other (about 2 cm) and performing HWT at 75 °C for 60 minutes (Fig. S3). During the experiment, we continuously and gently stirred the water to have a uniform laminar flow in the HWT container. SEM images of the Cu and Zn surface after HWT and corresponding EDS analysis, which are shown in Fig. S3, reveal the existence of ZnO nanowires not only on the Zn substrate, but also on Cu. In the process, as was revealed by Fourier transform infrared spectroscopy (FTIR) analysis on a water sample taken from the HWT experiment, ZnO molecules migrated through the water and re-deposited on the Cu surface. This further supports the important contribution of the migration step as part of the plugging mechanism in the formation of MONSTRs during HWT.

As for the surface diffusion step, mobility of the re-deposited oxide molecules are expected to depend on surface energy of the crystal facets that they land on. Their surface diffusion will be higher on the low surface energy planes that drives re-deposited molecules to move towards the edges and further extends size of the facet. Therefore, low surface energy crystal planes can grow faster and determine the final shape of the MONSTR. For this purpose, we first identified the crystal structures of MONSTRs based on XRD analysis (Figs S1 and S5), most of which are thermodynamically stable oxide phases. We then divided nanostructure shapes into four different categories including spherical, wire-like (e.g. wires and rods), 2D polygonal (leaf-like shapes such as plates, disks, and sheets), and 3D polygonal (e.g. cubes and pyramids) geometries. Bar diagram in Fig. S5 compares the relation between crystal structure and MONSTR shape. It is observed that most of the monoclinic, tetragonal, and orthorhombic oxides result in 2D polygonal nanostructure shapes. This indicates a dominant surface diffusion rate on a prominently low-surface energy crystal plane for these crystal structures. On the other hand, there does not seem to be a correlation between nanostructure shape and crystal structure for the cubic and hexagonal oxides we studied. This might be due to the competing and more comparable surface diffusion rates on different crystal planes of cubic and hexagonal metal oxides.

In addition, we performed HWT on a roughened Zn plate by sandblasting and compared the results to those on a relatively smooth polished Zn. As shown in Fig. S6, we observed that ZnO MONSTRs preferentially grew on the tops of the hills, while the growth was much uniform on the polished plate. This might be due to relatively long mean-free-path of ZnO molecules that can depend on several factors including their concentration in the water. Molecular oxide concentration can decrease at later stages of the HWT growth because of the limited supply of metal ions blocked by the oxide formed at earlier stages. For example, based on FTIR measurements (Fig. S3) on the HWT solution sample from later stages of the ZnO growth, we observed low concentrations of ~3.28 × 10^−12^ mol/cm^3^ that corresponds to an approximate distance of ~0.4 μm among ZnO molecules. Such a separation among ZnO molecules is comparable to the micrometer-to-submicrometer-scale surface features on the rough Zn substrate. Therefore, long mean-free-path of oxide molecules can lead to preferential deposition on hill-tops instead of valleys due to a “shadowing effect”, which is observed in physical vapor deposited thin films and nanostructures^54, 55^.

It is also important to keep the balance between the water temperature and the dissolved oxygen during HWT. Although at higher water temperatures, the rate of metal cations being released to water increases due to the enhanced surface activation, on the other hand, the dissolved oxygen levels decrease. This can then lead to a reduction in hydroxide ions levels due to lower oxygen concentration. Therefore, the formation of metal oxide molecules and MONSTRs might be limited at relatively high water temperatures. In other words, higher HWT temperatures can be desirable to activate the metal surface to form cations; yet, it does not necessarily mean that metal oxide nanostructures will form faster compared to lower temperatures. This might require the need for additional oxygen by means such as O_2_ purging into the water in order to increase MONSTR growth rates. In addition, we also performed experiments with the lid of the HWT beaker closed to isolate the water from ambient air and intentionally reduce the dissolved oxygen levels. We observed that MONSTR formation significantly reduced which further shows the importance of the metal oxide formation step in the proposed growth mechanisms described above.

Another factor that can affect the cation formation is the surface state that can be enhanced by means such as acid pre-treatment. For example, we treated Cu with nitric acid (HNO_3_) prior to HWT and observed that the critical time of MONSTR formation decreased from 24 hrs down to 8 hrs. In addition, surface pre-treatment can increase dangling bonds that can enhance the re-deposition step in the MONSTR growth mechanism (Fig. 4). For some materials, simple pre-treatment methods like mechanical sanding might be sufficient to activate the surface. We observed a notable increase in the MONSTR density on the surface of Zn when the base metal was sandblasted before the HWT. On the other hand, cation formation can be hindered due to impurities either in the bulk or at the surface of the base metal. Such impurities can act as inert species toward HWT and prevent the reaction of water with the underlying metal, or they can be more reactive than the base metal and form a protective oxide layer at the top.

Control of HWT-MONSTR properties such as size, geometry, separation, and crystal structure can potentially be achieved by changing the growth factors discussed above. For example, MONSTR size can simply be controlled by the HWT time up to a certain point where there is still supply of cations from the base metal. Geometry is strongly related to the crystal structure as discussed before. Therefore, the question can be how we can control the crystal structure. Parameters such as dissolved oxygen can affect the type of metal oxide phase and indirectly lead to different nanostructure shapes. Finally, separation can be controlled by the amount of substrate surface defects or through pre-existing surface bumps, on which MONSTRs can preferentially grow due to the shadowing effect.

We note that migration of metal oxide molecules through water might potentially raise health hazard concerns especially in situations where metals are in direct contact with hot water. In addition, due to the general growth mechanisms discussed above, MONSTRs can form on everyday metal tools even in the presence of unpurified water that can further increase the hazardous risks. For this purpose, we performed some preliminary tests on a copper pot that was used to heat tap water at 75 °C. The pot surface was polished before the experiment to mimic scrub-cleaning that removes pre-existing copper oxide layer and can activate the surface. Figure S7a shows the SEM image of copper oxide nanostructures that formed after 18 hours. Although not well developed, these MONSTRs can still come off from the surface of the pot when scrubbed again during the cleaning process, which can eventually contaminate water resources. Another example we looked into is an aluminum foil wrapped around hot food. The steam coming from the food can condense on the surface of Al foil, form a hot water droplet, and essentially create the conditions of MONSTR formation by HWT. Figure S7b shows the images of aluminum oxide nanostructures that emerged on the surface of an Al foil after being exposed to a steam source for 25 minutes. In such cases, it is quite likely that metal oxide molecules can get mixed into the heated water and consumed by humans or animals, which requires a detailed investigation.

## Conclusion

In conclusion, we presented a new hot water treatment to synthesize metal oxide nanostructures that is applicable to a wide range of metals including elemental ones as well as alloys and compounds. The HWT method is a simple, low-temperature, scalable, low-cost, and eco-friendly method that overcomes most of the limitations of other conventional synthesis techniques to produce MONSTRs. Therefore, this unique technique can pave way to the utilization of MONSTRs in several applications such as gas sensors, photodetectors, solar cells, supercapacitors, and batteries that can immensely benefit from high surface area metal oxides. HWT process simply includes the immersion of a metal piece into hot DI water without any chemical additives such as metal salt, reducing, or oxidizing agents. Out of the 45 different elemental metals, alloys, and compounds, 30 of them responded to HWT at temperatures either at 75 °C or 95 °C within time periods ranging from tens of minutes to several hours, which lead to the clear formation of MONSTRs on the metal surface. In addition, we investigated the growth mechanisms involved in the formation of MONSTRs during HWT. Our findings reveal that a plugging process that includes the release, migration, and re-deposition of metal oxide molecules through water is critical in addition to surface diffusion during the MONSTR growth. Furthermore, our preliminary tests show that HWT can also produce MONSTRs even in unpurified water such as tap water, for example on the surface of a copper cooking pot. We also observe that hot water droplets condensing from steam on the surface of a metal can form MONSTRs, for example, on aluminum foil wrapped around a steaming hot food. Therefore, there is a need for detailed research on the potential health hazards of metals interacting with hot water.

## Method

We studied the following elemental, alloy, and compound metals: Titanium (Ti), nickel (Ni), molybdenum (Mo), cadmium (Cd), zinc (Zn), tin (Sn), magnesium (Mg), lead (Pb), indium (In), copper (Cu), aluminum (Al), manganese (Mn), iron (Fe), cobalt (Co), scandium (Sc), neodymium (Nd), dysprosium (Dy), selenium (Se), vanadium (V), bismuth (Bi), zirconium (Zr), silver (Ag), silicon (Si), platinum (Pt), germanium (Ge), chromium (Cr), tantalum (Ta), gold (Au), thallium (Tl), niobium (Nb), beryllium (Be), yttrium (Y), hafnium (Hf), antimony (Sb), tungsten (W), erbium (Er), terbium (Tb), tellurium (Te), and gadolinium (Gd) with purity of 99.95%, Al alloys (#6061 and #5052), bronze (88% Cu and 12% Sn), brass (60% Cu and 40% Zn), stainless steel (#304), and plain steel. Some metals were excluded from this study because of their incompatibility with the HWT process, such as metals with low melting points < 50 °C, the ones in liquid and gas state in ambient conditions, metals that react explosively with water even at low temperatures, and those that are artificially synthesized. First, pieces of metal samples were polished with ultrafine sanding paper of 5000 grits to remove native oxides and other organic contaminations, then cleaned by ultra-sonication in acetone, isopropanol, and DI water each for 10 min. Samples were imaged by SEM to confirm planar nanostructure-free surfaces before the HWT process (Fig. S3). In addition, we characterized the substrate surface composition before HWT through EDS analysis (Table S2), which shows an elemental metal surface without any indication of notable surface contamination.

Then, cleaned metals were immersed in glass beakers filled with ultrapure hot DI water and placed on a hot plate (Fig. 4a), and were treated for time periods ranging from 10 min and up to 24 hrs. We defined the critical time of MONSTR formation (Fig. 2) as the period beyond which the geometry and density of nanostructures did not significantly change further. Finally, samples were dried with nitrogen and delivered for morphological, crystallography, and chemical composition analysis by SEM (JEOL SEM7000FE), XRD (Bruker D8-Discover), and EDS (EDAX), respectively. FTIR was performed on a water sample taken from the HWT experiment on Zn plates in order to study the presence of ZnO molecules in water. Based on our preliminary results on Al, Zn, and Cu, 75 °C was chosen to be the standard water temperature for the HWT experiments of this study. However, those metals that did not form well-defined MONSTRS at 75 °C were treated at a higher temperature of 95 °C. HWT process was performed at different durations of 10-min steps for 10–60 minutes, and if no nanostructures were observed then treatment time was extended to hours scale with 2-hour steps for 2–6 hrs and 4-hour steps for 8–24 hrs.

## Electronic supplementary material

Supplementary Information

Hydrogen gas formation in HWT experiments
