# Supplementary material for: Metal oxide nanostructures by a simple hot water treatment
Source: Sci Rep. 2017 Aug 2;7:7158. doi: 10.1038/s41598-017-07783-8 (PMC5541048; doi:10.1038/s41598-017-07783-8)
Supplement: Supplementary file 1 — Supplementary Information [file 41598_2017_7783_MOESM1_ESM.pdf]

Metal oxide nanostructures by a simple hot water treatment: Supplementary information

Nawzat S. Saadi*, Laylan B. Hassan & Tansel Karabacak*

Department of Physics and Astronomy, University of Arkansas at Little Rock, 2801 South University Avenue, Little Rock, AR 72204, USA

Table S1 Expected metal oxide compositions and measured EDS atomic compositions

Base Metal	Expected Oxide State	Top-View SEM	Atomic Composition from EDS		Base Metal	Expected Oxide State	Top-View SEM	Atomic Composition from EDS		Base Metal	Expected Oxide State	Top-View SEM	Atomic Composition from EDS	
			Metal %	Oxygen %				Metal %	Oxygen %				Metal %	Oxygen %
Al	Al_2O_3	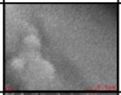	53	47	Ta	TaO	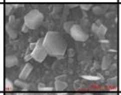	56	44	Ni	NiO	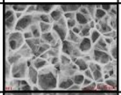	54	46
Mg	MgO	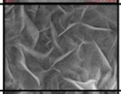	62	38	Fe	Fe_2O_3	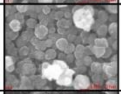	80	20	In	In_2O_3	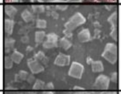	39	61
Zn	ZnO	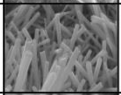	66	34	Se	SeO_2	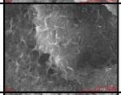	84	16	V	VO	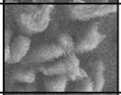	75	25
Cu	CuO	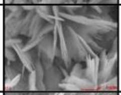	60	40	Nd	Nd_2O_3.	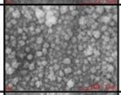	34	66	Ti	TiO_2	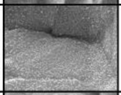	60	40
Cd	CdO	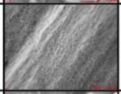	53	47	Tl	Tl_2O	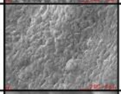	63	37	Be	BeO	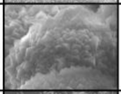	below detection limit	
Mo	MoO	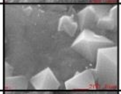	64	46	Pb	PbO	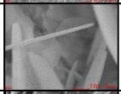	65	35	Er	Er_2O_3	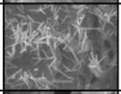	33	67
Sc	ScO	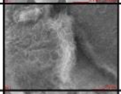	66	34	Co	CoO	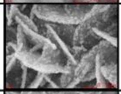	54	46	Sb	Sb_2O_3	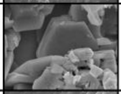	35	65
Mn	MnO	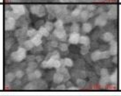	75	25	Bi	Bi_2O_3	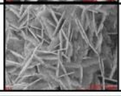	37	63	Sn	SnO	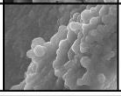	62	38

Supplementary Table 1. The table lists expected energetically stable metal oxide compositions of this study and atomic compositions measured from EDS on the selected MONSTRs shown in the top-view SEM images. (Note: EDS might have picked up signal from the base metal substrate, especially for samples of thin MONSTR layers, which overestimates metal atomic composition for some of the samples.)

Table S2 EDS Atomic compositions of metal surface before HWT

Sample	Atomic Composition from EDS		Sample	Atomic Composition from EDS		Sample	Atomic Composition from EDS	
	Base Metal %	Other %		Base Metal %	Other %		Base Metal %	Other %
Al	100	0	Ta	100	0	Ni	100	0
Mg	100	0	Fe	97	C:3	In	100	0
Zn	100	0	Cr	98	C:2	V	99	C:1
Cu	100	0	Nd	100	0	Ti	96	C:4
Cd	95	C:5	Tl	100	0	Be	below detection limit	0
Mo	99	C:1	Pb	97	C:3	Er	100	0
Sc	100	0	Co	100	0	Sb	100	0
Mn	100	0	Bi	100	0	Sn	98	C:2

Supplementary Table 2. Atomic composition of elemental base metals of this study measured by EDS analysis on the samples after polishing and before HWT.

Figure S1 XRD profiles of HWT metal oxide nanostructures

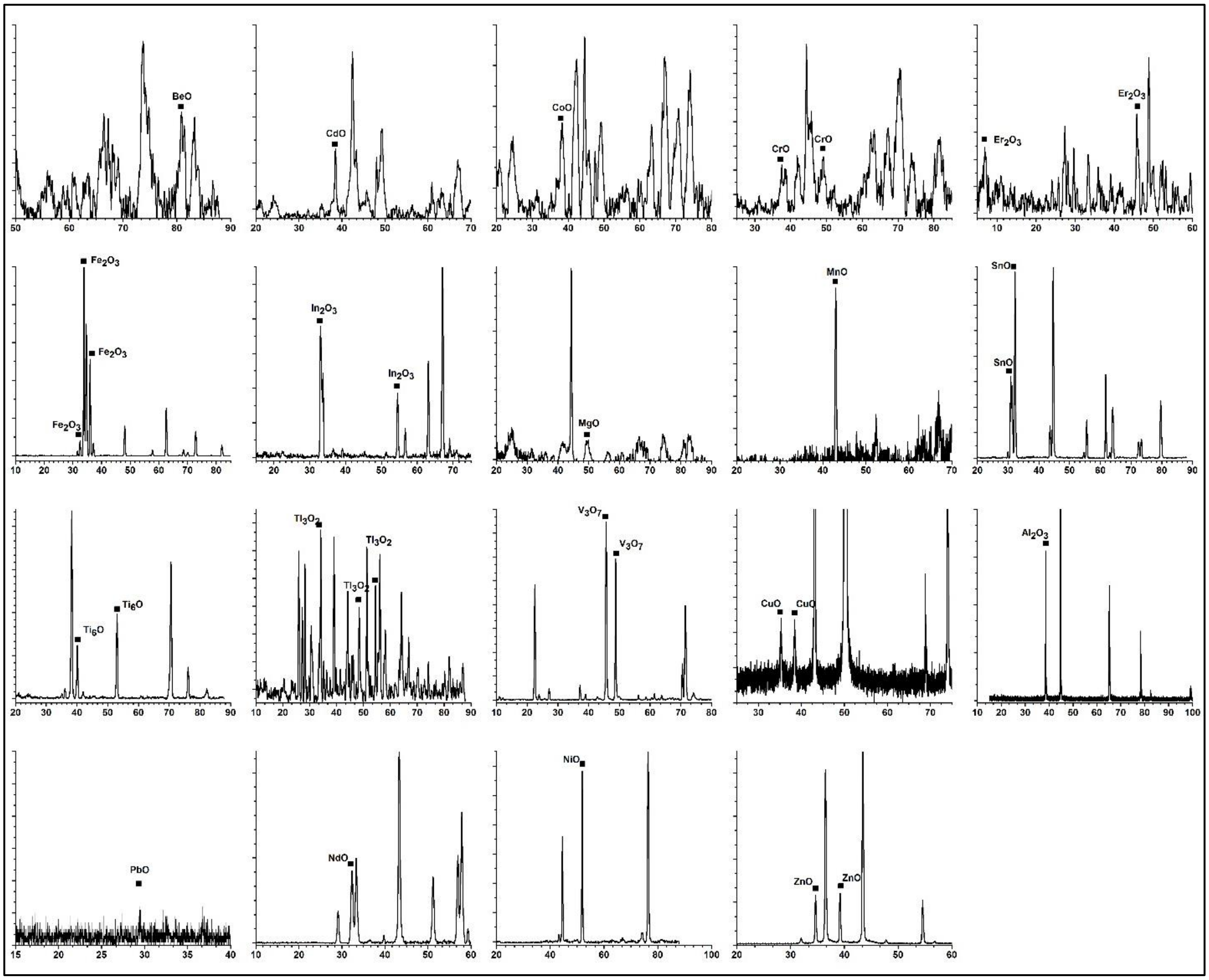

Supplementary Figure 1. θ-2θ XRD profiles of the most dominant oxide peaks observed for the metals that responded to HWT and formed MONSTRs. Few other metals also formed MONSTRs but did not show any notable XRD peak due to the amorphous structure and/or not sufficiently thick oxide formation.

Figure S2 Low magnification SEM images of some selected metals

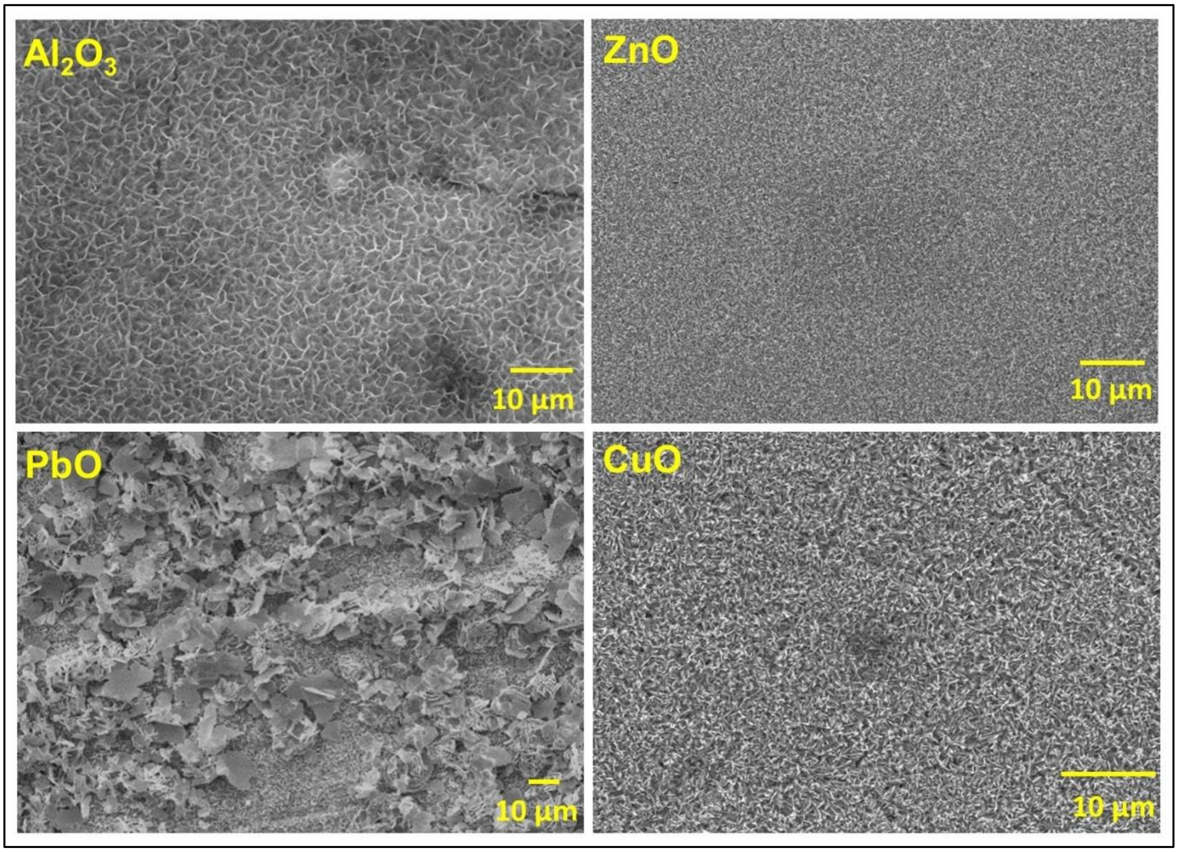

Supplementary Figure 2*.* Lower magnification top-view SEM images of some of the selected metals show the uniform coverage of their surface with crystalline MONSTRs after HWT. The metals selected include highly-reactive (Al, Pb, and Zn) and relatively slow-reacting (Cu) metals to HWT.

Figure S3 Plugging mechanism during HWT process

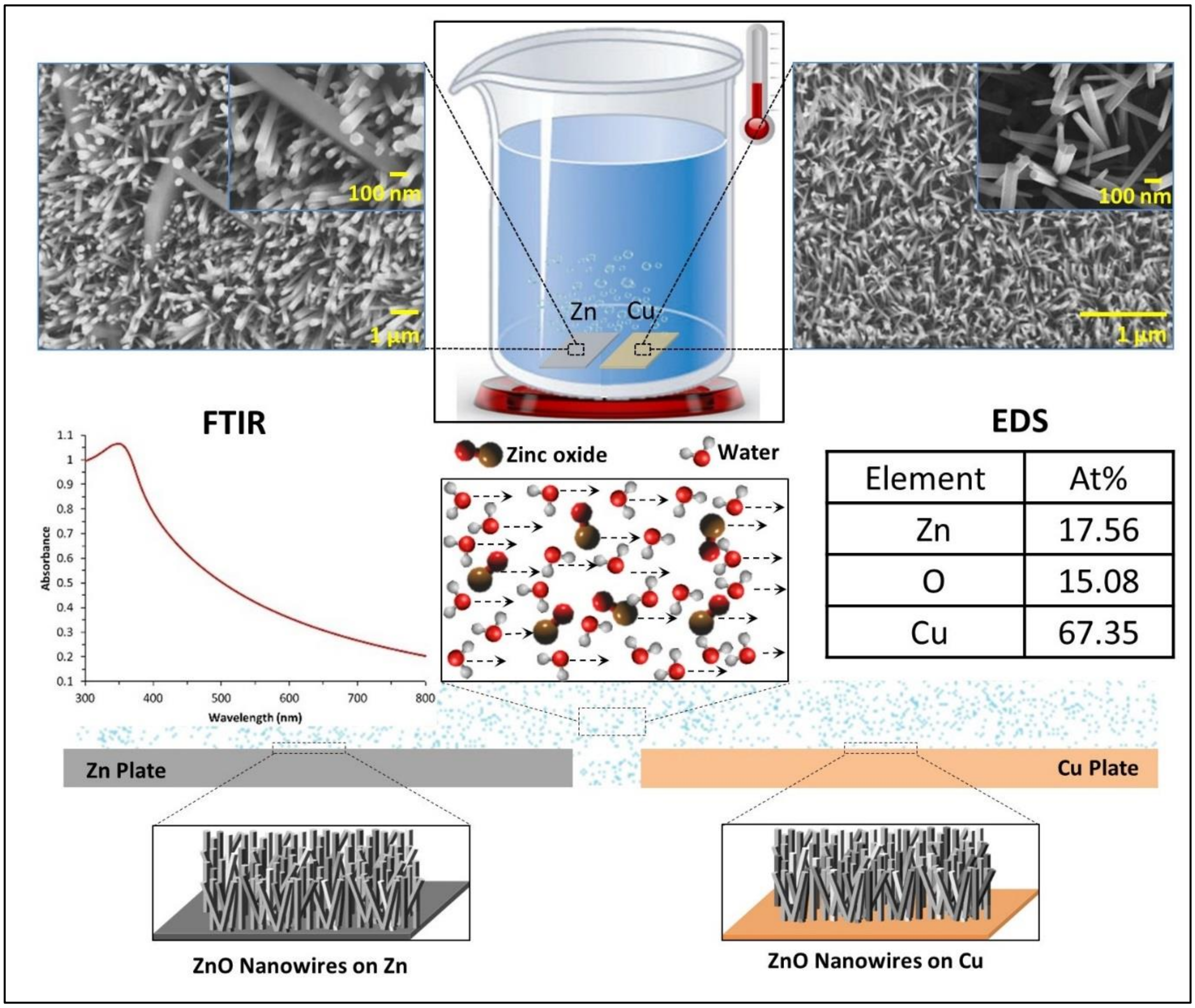

Supplementary Figure 3. A HWT experiment with Zn (left) and Cu (right) plates being treated together shows that ZnO nanorods form on the surface of copper substrate (top-view SEM image on the right), which reveal the existence of "plugging" mechanism. FTIR profile of a sample solution taken from the water during HWT also shows presence of ZnO molecules that migrates from Zn towards Cu plate. Atomic compositions measured from EDS on the Cu surface show the existence of Zn.

Figure S4 Metal surface after the cleaning process and before HWT

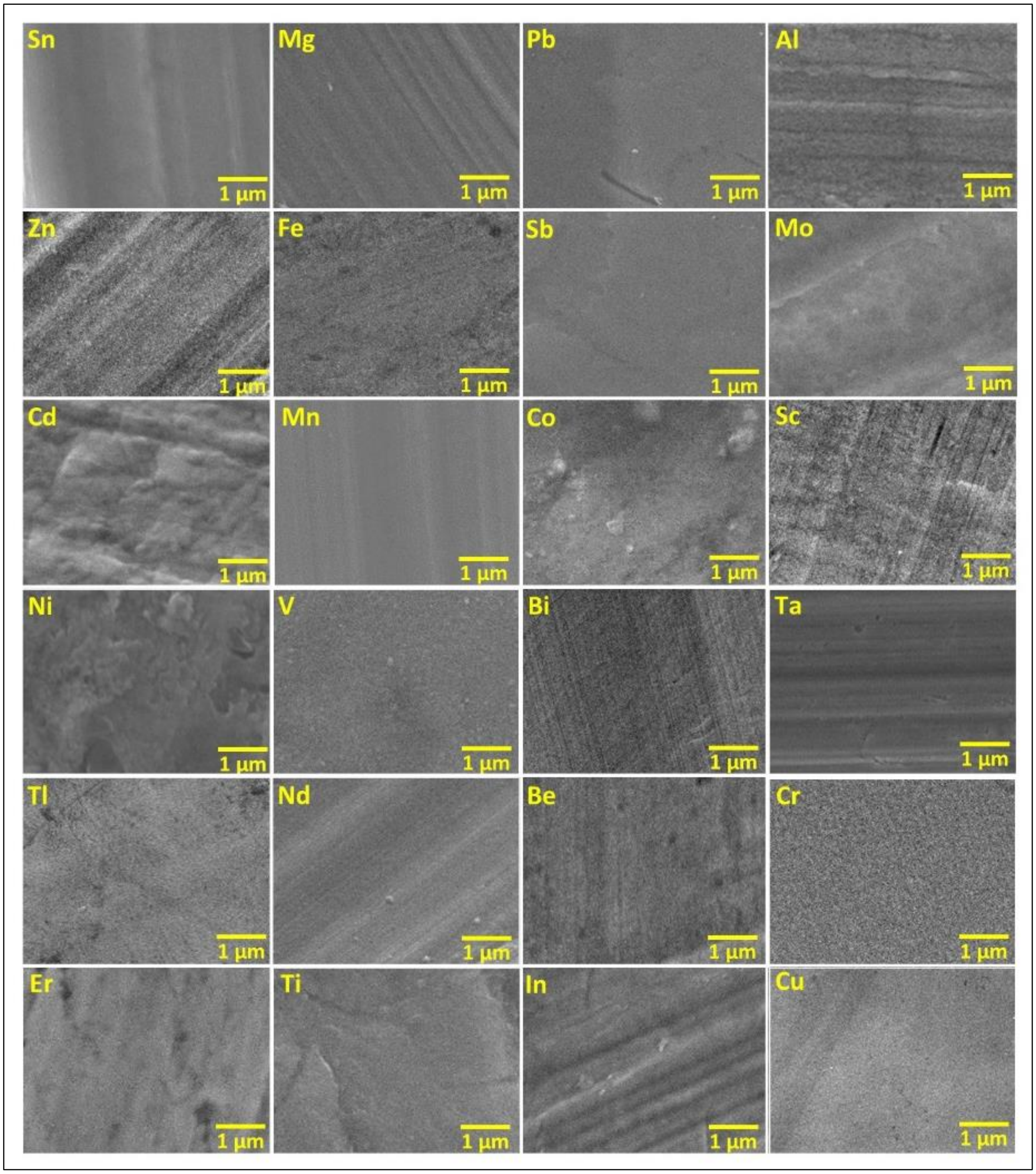

Supplementary Figure 4. Top-view SEM images metal surfaces before HWT.

Figure S5 HWT-MONSTRs shapes and their oxide crystal structure

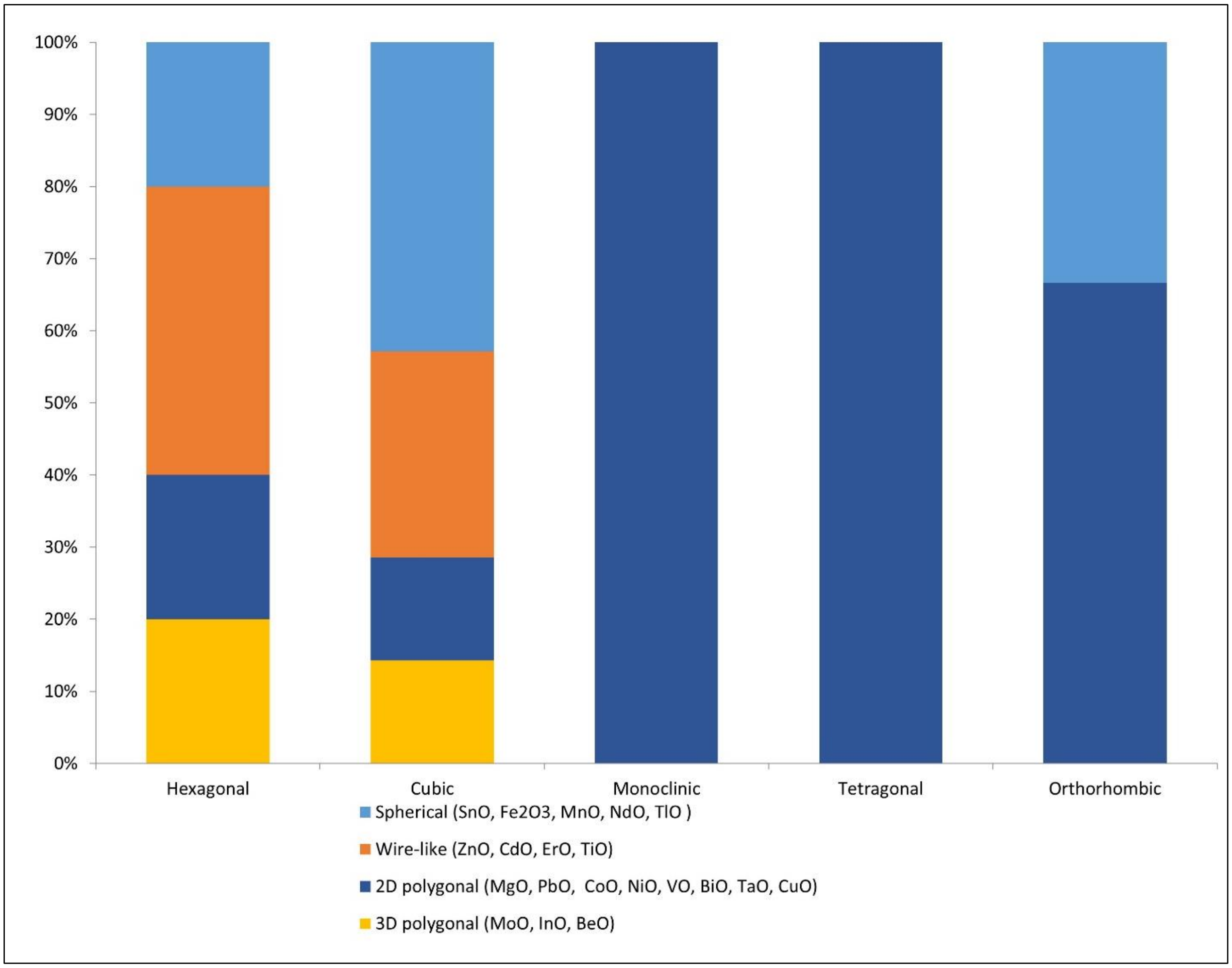

Supplementary Figure 5 Percentage distribution of basic nanostructure shapes depending on the crystal structure of MONSTRs.

Figure S6 ZnO MONSTRs on rough Zn plate

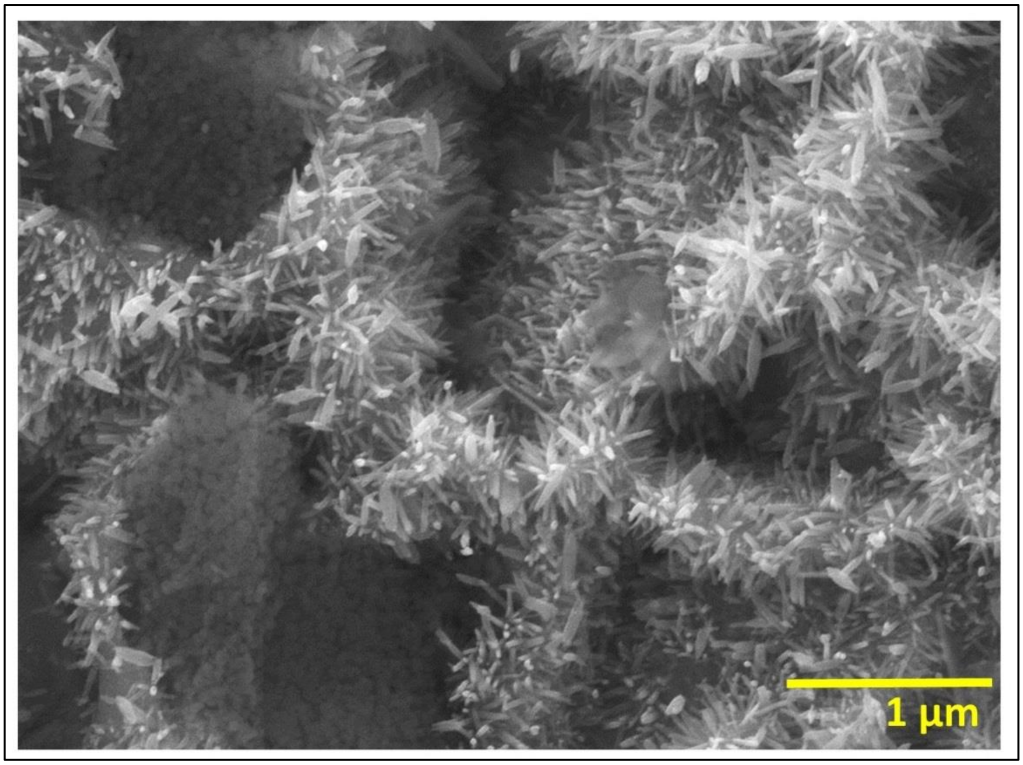

Supplementary Figure 6 Top-view SEM image of HWT ZnO MONSTRs that formed on a roughened Zn plate. The image shows that ZnO nanostructures preferentially grew on the hill-tops of the rough substrate.

Figure S7 MONSTRs formation on Cu pot surface by HWT using unpurified water and aluminum foil exposed to a steam source

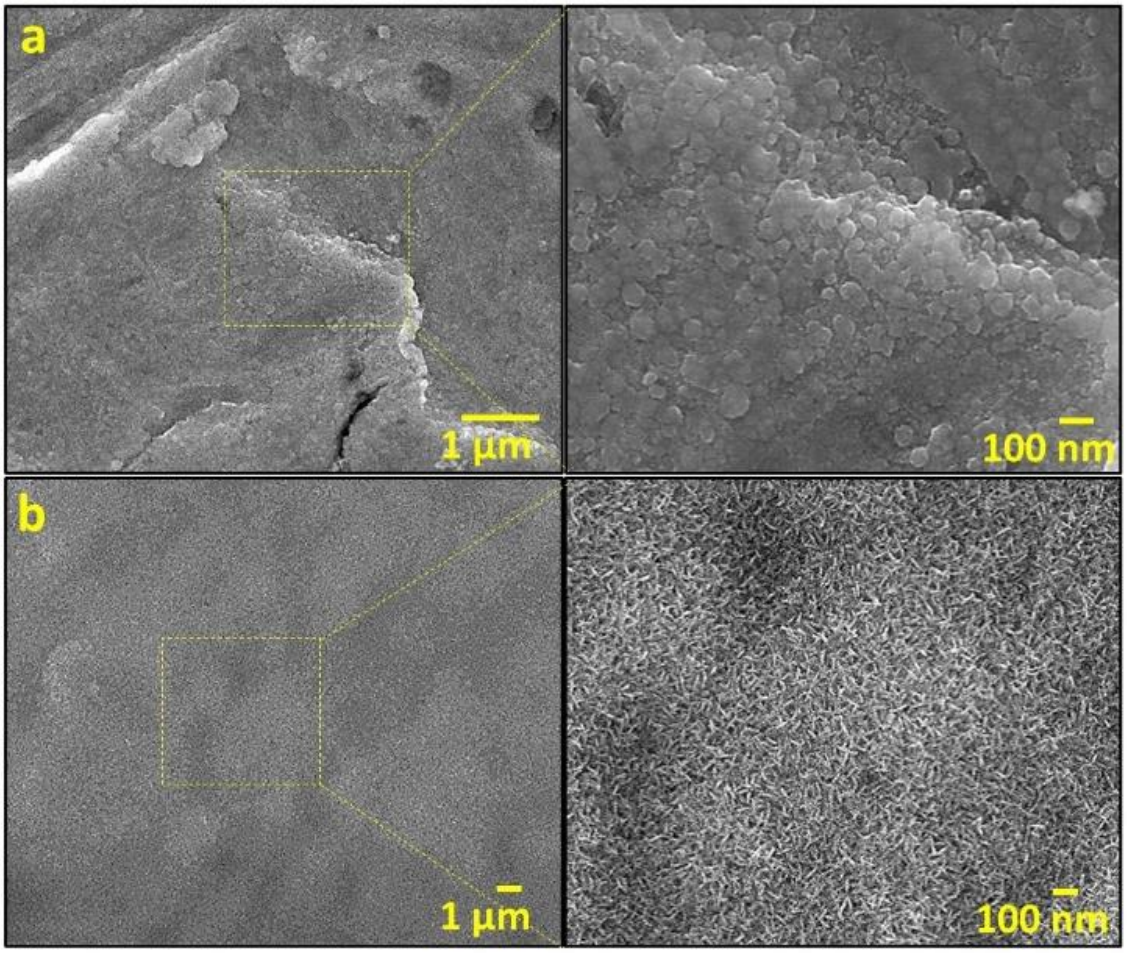

Supplementary Figure 7 (a) HWT experiment with Cu pot using unpurified water and (b) Al foil being exposed to a steam source. Top-view SEM images on the right reveal the formation of MONSTRs on both Cu and Al surface.
